# Structural and functional abnormalities of vision-related brain regions in cirrhotic patients: a MRI study

**DOI:** 10.1007/s00234-019-02199-9

**Published:** 2019-04-04

**Authors:** Mingquan Wang, Jin Cui, Yanpeng Liu, Yawen Zhou, Huijuan Wang, Yanming Wang, Yuying Zhu, Benedictor Alexander Nguchu, Bensheng Qiu, Xiaoxiao Wang, Yongqiang Yu

**Affiliations:** 1 Department of Radiology, The First Affiliated Hospital of AnHui Medical University, Hefei, 230022 Anhui China; 2 Centers for Biomedical Engineering, University of Science and Technology of China, Hefei, Anhui China

**Keywords:** Functional MRI, Hepatitis B virus–related cirrhosis, Vision-related regions, Voxel-based morphometry

## Abstract

**Purpose:**

Previous studies have focused on global cerebral alterations observed in cirrhosis. However, little was known about the specific abnormalities of vision-related brain regions in cirrhotic patients. In this study, we sought to explore neurological alterations of vision-related regions by measuring brain resting-state network connectivity, based on the structural investigation in cirrhotic patients without clinical sign of hepatic encephalopathy (HE).

**Methods:**

Structural and functional magnetic resonance image (MRI) data were collected from 20 hepatitis B virus (HBV)-related cirrhotic patients without clinical sign of HE and from 20 healthy controls (HC). Voxel-based morphometric (VBM) analysis and brain functional network analysis were performed to detect abnormalities in cerebral structure and function.

**Results:**

Cirrhotic patients showed regions with the most significant gray matter reduction primarily in vision-related brain regions, including the bilateral lingual gyri, left putamen, right fusiform gyrus, and right calcarine gyrus, and other significant gray matter reductions were distributed in bilateral hippocampus. Based on structural investigation focused on vision-related regions, brain functional network analysis revealed decreased functional connectivity between brain functional networks within vision-related regions (primary visual network (PVN), higher visual network (HVN), visuospatial network (VSN)) in the patient group compared with HC group.

**Conclusion:**

These results indicate that structural and functional impairment were evident in the vision-related brain regions in cirrhotic patients without clinical sign of hepatic encephalopathy. The physiopathology and clinical relevance of these changes could not be ascertained from the present study, which provided a basis for further evolution of the disease.

## Introduction

Hepatitis B virus–related cirrhosis is a global public health problem characterized with high infection, morbidity, and mortality rates [1]. It has been estimated that the chronic HBV infection affects 350 million people worldwide (more than 5% of the world population), who suffer from brain edema, intracranial hypertension, and widespread cerebral neurological deficits [2–4].

In some case reports regards cirrhosis, the vision-related regions were shown to be affected, which lead to transient losses of the vision [5–8]. Zafiris et al. have also reported that cirrhotic patients without clinically overt hepatic encephalopathy (HE) showed impaired performance in vision capacity tasks with reference to the extrastriate visual cortex [9]. Cirrhotic studies based on MRI, a noninvasive imaging technique, have repeatedly reported the neuroimaging changes within different vision-related regions. For instance, MRI studies showed that cirrhotic patients exhibited a significant reduction in gray matter regions, particularly in putamen, fusiform gyrus, and occipital regions [10–12]. A neuroimaging study has reported the occipital-parietal cortical edema by MRI and focal occipital status epilepticus by electroencephalogram (EEG) in the HBV-related hepatic disease [8]. Reduced cortical thickness was observed in the occipital cortex in patients with hepatitis C virus infection [13]. In this study, we sought to study alterations in cerebral gray matter volume in cirrhotic patients by voxel-based morphometric analysis. This type of analysis permitted an unbiased general search of structural abnormalities across the entire brain. Based on this structural investigation, the resting-state functional MRI (rs-fMRI) was further performed to detect abnormalities within these regions.

The rs-fMRI measured spontaneous brain activity as low-frequency fluctuations in blood oxygen level-dependent (BOLD) signals. During the resting-state, correlated spontaneous fluctuations occurred within spatially distinct and functionally related groups of cerebral regions, in which variations would reflect task performances in the real life [14]. Furthermore, the resting-state method has been used to reveal functional architecture in the brain of cirrhotic patients and could serve as a marker to reflect altered features of cirrhosis without overt HE, particularly in lingual gyrus, middle occipital gyri, and cuneus [15–17]. It was worth noting that the vision-related brain regions were a complex patchwork of functionally interconnected regions, and previous studies have not adequately provided a systematic investigation into aberrant organization among these regions.

The method of brain functional network analysis could provide a new way of understanding human brain function procedures and investigating dysfunctional brain architecture in cerebral alterations [18, 19]. The systematic investigation of distinct brain functional networks could provide an important perspective to uncover mechanisms regarding brain alterations [20]. To the author’s knowledge, there was not any MRI study having examined alterations focused on vision-related regions using brain functional network analysis, which would provide a systematic insight into the vision-related regions from a more comprehensive perspective in cirrhotic patients. Thus, brain functional network analysis would be a useful method to furtherly quantify disease-related pathophysiological changes within these regions.

Therefore, we hypothesized that cirrhotic patients would exhibit abnormalities in the vision-related brain regions compared to HC group. From a systematic perspective by neuroimaging investigations, this study aimed to explore how vision-related regions were affected in the cirrhotic group without clinical sign of overt HE.

## Methods

### Participants

This study was approved by the local Research Ethics Committee. Written informed consents were obtained from all the participants before the study. The clinical and demographic data are shown in Table 1.

**Table 1:** Demographics and clinical characteristics of cirrhosis patients and healthy controls

Variable	Cirrhosis group (*n* = 20)	HC group (*n* = 20)	*p* value
Age (years)	51.65 ± 11.25	51.00 ± 10.13	0.85^a^
Sex ratio (M/F)	11/9	14/6	0.18^b^
Venous blood ammonia (in μ mol/L)	51.60 ± 33.34	N/A	
Child-pugh stage (A/B/C)	4/4/7	N/A	
Album	34.30 ± 4.75	N/A	
Total serum bilirubin	20.73 ± 9.84	N/A	
Prothrombin time	38.78 ± 9.24	N/A	

Values are expressed as mean ± SD

^a^The *p* value for age difference between the two groups was obtained by two sample *t* test

^b^The *p* value for gender distribution in the two groups was obtained by chi-square test

We employed 20 HBV-related cirrhotic patients without clinical sign of HE for participation in the study. Considering the needs of clinical treatment, no clinical sign of HE was diagnosed during recruitment by the two professional physicians in the local hospital. Patients were excluded if they showed or were judged to exhibit current symptoms of overt HE at the time of recruitment, other types of viral hepatitis, a transjugular intrahepatic portosystemic or a surgical portacaval shut, or any history of alcohol abuse. Laboratory indices including venous blood ammonia, albumin, total serum bilirubin, and prothrombin time were obtained from patients for the assessment of the functional state of the liver. For comparisons, 20 age and sex-matched healthy controls were recruited from the hospital. Exclusion criteria for healthy controls included less than 5 years of formal education, false teeth, or clinically determined diseases of the liver or other systems.

### Imaging acquisition

All MRI images were collected from a GE Signa HDxt 3.0T MRI scanner (GE Medical Systems, Milwaukee, WI) equipped with a standard eight-channel head coil. Gradient specifications: amplitude 50 mT/m, slew rate 150 T/m/s. The resting-state functional MRI images were recorded with the following settings: repetition time/echo time, 2000/30 ms; flip angle, 90°; slice thickness, 3 mm; matrix size, 64 × 64; and field of view, 240 mm × 240 mm and fMRI volumes, 240 (the first four fMRI volumes were removed before analysis). The T1-weighted anatomic images were acquired in sagittal orientation with three-dimensional inversion recovery prepared fast spoiled gradient recalled sequence with following parameters: repetition time/echo time radio, 7.012/2.876 ms; inversion time, 90 ms; flip angle, 8°; field of view, 256 mm × 256 mm; slice thickness, 1.2 mm; voxel size, 1 mm × 1 mm × 1 mm; and the number of slices, 166. All the patients and healthy controls were examined in resting state with closed eyes.

### Image preprocessing

The fMRI data were preprocessed by using Analysis of Functional NeuroImages (AFNI) software tools (Medical College of Wisconsin, Milwaukee, WI, USA) and FSL (the FMRIB Software Library, Oxford, UK). First, structural and functional images were reoriented to MNI standard orientation. Then, skull stripping and motion correction were performed. Next, the individual structural images were registered to the Montreal Neurological Institute (MNI) standard template with a resolution of 1 mm × 1 mm × 1 mm using 12 degrees of freedom of the affine transformation (FSL flirt) and non-linear transformation (FSL flirt, optimizing the local deformations), and transformation file is generated simultaneously. After that, the individual functional images were linearly registered to the individual structural images by the rigid body of six degrees of freedom. Then, functional images were registered to the MNI space using the transformation file which was generated before. The motion was also calculated and data with head motion over 2 mm or 2° were excluded (2 mm or 2° is a common criterion for excluding the head motion) [21–23], and all the fMRI data met the criteria. To remove low-frequency drift and high-frequency noises, all fMRI signals were filtered by band-pass filtering (0.01–0.08 Hz) and then spatially smoothened using a 6-mm full width at half maximum Gaussian kernel. In addition, because of the topic focused on the human cerebrum, the cerebellum was removed. After being preprocessed, the individual data were used for further correlation analyses.

### VBM analysis

Structural data was analyzed using FSL-VBM (http://fsl.fmrib.ox.ac.uk/fsl/fslwiki/FSLVBM), an optimized VBM protocol included in FSL tools. First, structural images were brain-extracted and gray matter-segmented before being registered to the MNI152 standard space using non-linear registration. The resulting images were averaged and flipped along the *x*-axis to create a left-right symmetric, study-specific gray matter template. Second, all native gray matter images were non-linearly registered to this study-specific template and “modulated” to correct for local expansion (or contraction) due to the non-linear component of the spatial transformation. The modulated gray matter images were then smoothed out with an isotropic Gaussian kernel with a sigma of 3 mm. Then, voxel-wise GLM was applied using permutation-based non-parametric testing, and VBM results were corrected for multiple comparisons using the threshold-free cluster enhancement (TFCE) method with family-wise error (FWE) across space. Results were considered to be significant for *p* < 0.0002 after FWE correction.

### Mask ICA (mICA) and identification of resting-state networks (RSN)

Independent component analysis (ICA) is a widely used technique for studying functional connectivity (FC) in fMRI data. The mask independent component analysis (mICA), restricted to a defined region of interest, has been shown to detect local FC networks in particular brain regions [24].

Using the mICA toolbox, the components to be retained for further analysis were selected on the basis of the resting-state atlas template defined by Richiardi et al. [25]. In these templates, vision-related networks included primary visual network (PVN, 2 ROIs), higher visual network (HVN, 4 ROIs), and visuospatial network (VSN, 13 ROIs). The VSN included mainly bilateral precentral gyrus, bilateral postcentral gyrus, and bilateral superior/left inferior temporal gyrus. The HVN consisted mainly of right occipital lobe and left middle occipital gyrus. The PVN was made up primarily of the left lingual gyrus and bilateral calcarine gyrus.

### Network functional connectivity analysis

For each participant, the mean of time series was extracted from each ROI, and pairwise ROI functional connectivity was then calculated as the Pearson correlation between perspective mean time series [26]. The functional connectivity within (intra-FC) and between (inter-FC) networks were averaged across pairwise ROI functional connectivity [27], respectively, within single network and between every pair of network. This was used to generate a 3 × 3 network correlation matrix for each participant. Then mean network connectivity matrices were averaged across participants after Fisher’s r-to-z transformation [28]. The network functional connectivity was analyzed using the scripts in Matlab (MathWorks, Natick, MA).

The Fisher’s r-to-z transformation was conducted in order to determine the statistical differences in network functional connectivity between the patients and HC group. To reduce the probability of type I error, we controlled the false-discovery rate (FDR, *p* < 0.05) for comparisons within each subsystem.

### Correlation analysis

Pearson correlation and liner regression modeling were performed to assess the relationship between the functional changes and biochemical parameters (i.e., blood ammonia, total serum bilirubin). The statistical significant was set at *p* < 0.05.

## Results

**Fig. 1:**
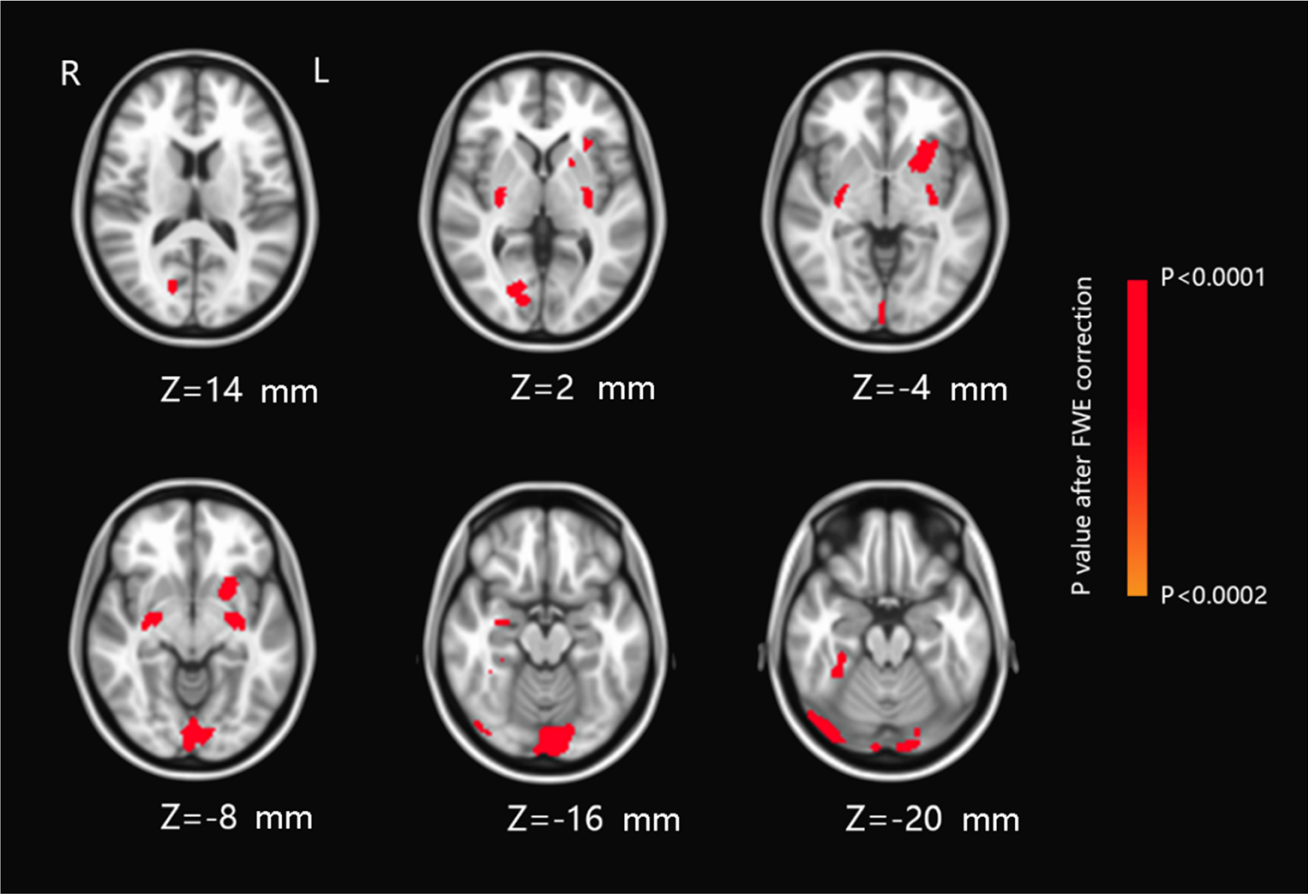
VBM analysis results between cirrhotic patient group and healthy controls group. Significant gray matter deficits were detected in right lingual gyrus, right fusiform, right calcarine gyrus, left putamen, and bilateral hippocampus. Significant thresholds were set at a *p* < 0.0002 after FWE correction. VBM, voxel-based morphometric; FWE, family-wise error

There were no significant differences (*p* > 0.05) between the cirrhotic group and healthy group in age and sex (Table 1). VBM analysis, based on the TFCE methods with FWE correction, showed the specific pattern of gray matter deficits that we have identified in cirrhotic patients (Fig. 1). Gray matter volume decreased in patient group was detected in the following areas: right lingual gyrus, right fusiform, and right calcarine gyrus compared with HC group. In addition, several subcortical structures, including left putamen and bilateral hippocampus, also showed marked volume reduction among the patients. Table 2 provides an overview of detectable structural brain changes of cirrhotic patients compared with HCs.

**Table 2:** Group-wise VBM comparison of the significant clusters showing reduced gray matter volume in patient group compared with healthy control group

Cluster size (voxels)	MNI coordinates			Region	*p* value (peak)
	*X* (mm)	*Y* (mm)	*Z* (mm)		
916	20	− 96	− 24	Right lingual gyrus	*p* < 0.0002
294	− 24	10	− 10	Left putamen	*p* < 0.0002
270	32	− 8	− 18	Right hippocampus	*p* < 0.0002
230	− 26	− 10	− 14	Left hippocampus	*p*< 0.0002
148	24	− 72	− 2	Right lingual gyrus	*p* < 0.0002
77	36	− 40	− 26	Right fusiform gyrus	*p* < 0.0002
51	16	− 76	12	Right calcarine gyrus	*p* < 0.0002

Threshold at a cluster significance level at *p* < 0.0002 after FWE correction. The automated anatomical labeling (AAL) atlas was used to label each cluster [29]

**Fig. 2:**
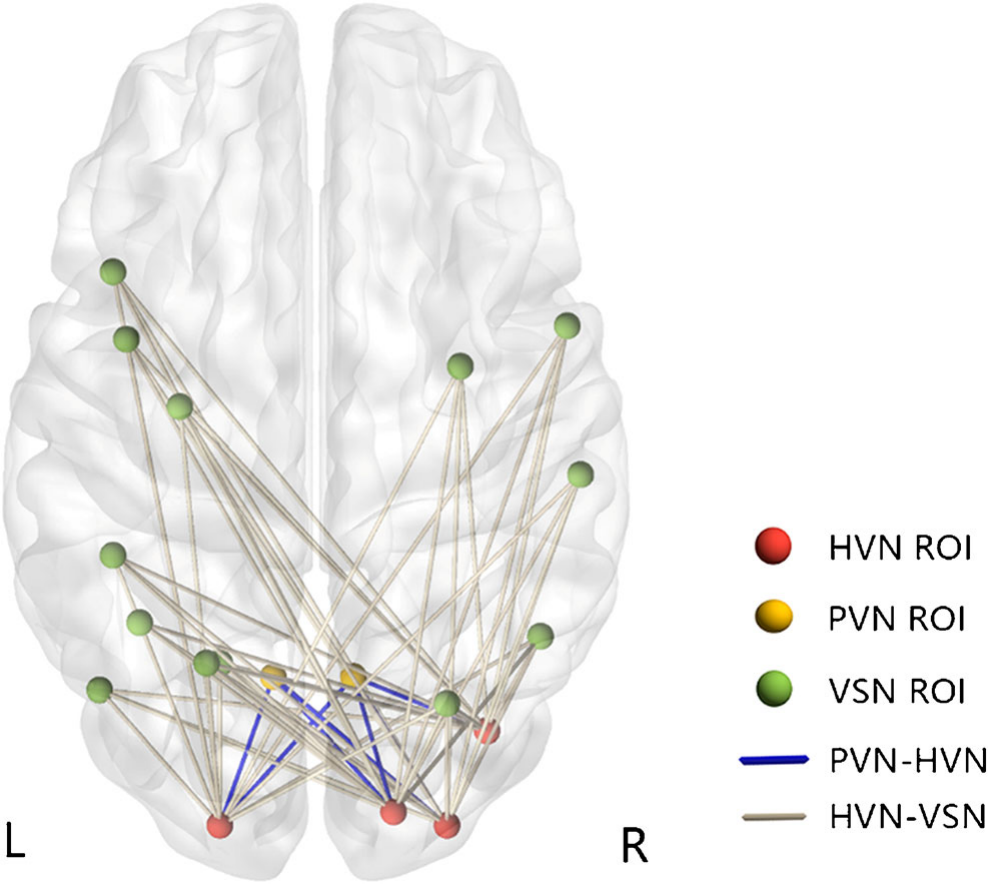
Decreased functional connectivity between vision-related networks. Brain graph showing the network connectivity of nodes (ROIs of vision-related networks) via edges (line, blue for connectivity between HVN and PVN, white for connectivity between HVN and VSN), indicates the decreased connections between HVN and PVN/VSN respectively

**Fig. 3:**
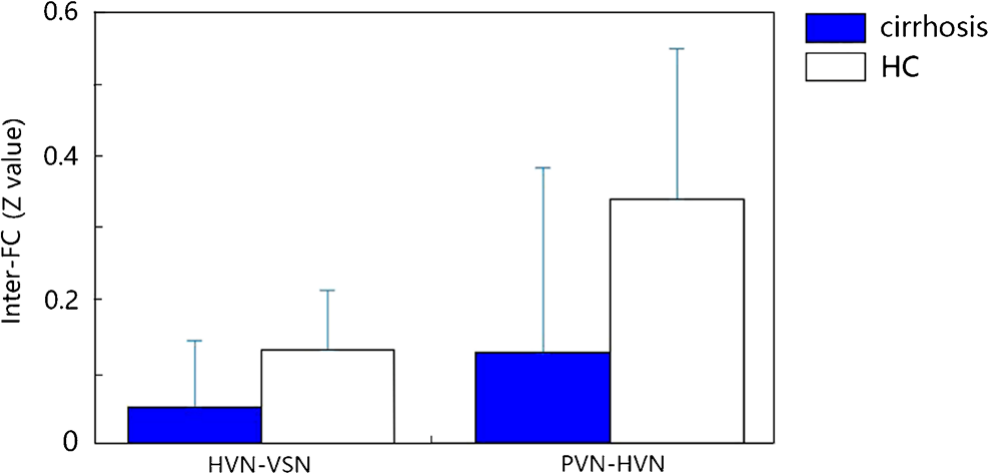
Statistical differences of FC between the patients group and HC group. The vertical axis is inter-FC strength (mean *Z* value) between HVN and VSN, as well as between HVN and PVN. The horizontal axis is pair networks with significant different connectivity coefficient. (Error bar indicates the st.d.). Between-group differences of inter-networks have been corrected by FDR

**Fig. 4:**
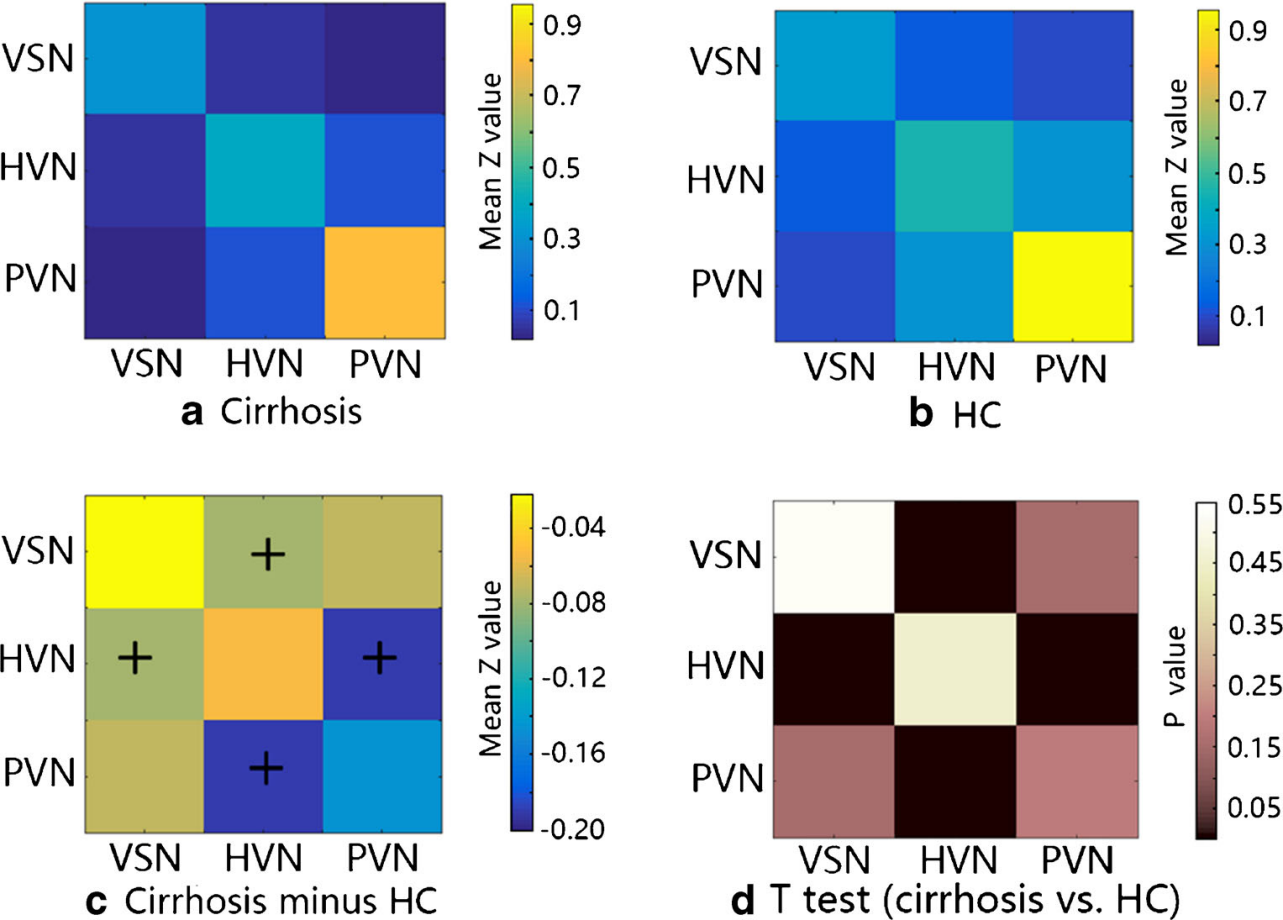
Vision-related networks average functional connectivity matrices. Pairwise Pearson’s correlations between the time courses were Fisher Z-transformed and averaged across subjects for each group. **a** HBV-related cirrhotic patients, **b** healthy control. The intensity of colors in the matrix (**a**) and (**b**) indicates the strength of pairwise network FC. **c** Patients minus HC, colors of the grid in the matrix C represent the differences of pairwise network FC. The marker + indicates a significant difference between two groups (*p* < 0.05 after FDR). **d** The statistical results by *t* test

By resting-state brain functional network analysis, cirrhotic patients showed significantly decreased FC between PVN and HVN, and between VSN and HVN compared with HCs (Figs. 2 and 3). No significant difference was observed in the intra-network connections*.* Figure 4 and Table 3 show the values of pairwise network FC strength on the network connectivity matrix for patients and HCs respectively. There was not any significant correlation between the functional changes and biochemical parameters (i.e., blood ammonia, total serum bilirubin) following correlation analysis.

**Table 3:** Group-wise comparison of pairwise FC between vision-related networks

	Patients (mean ± st.d.)	HC (mean ± st.d.)	*p* value (*t* test)
Intra-FC			
VSN	0.3092 ± 0.0825	0.3293 ± 0.1160	0.5326
HVN	0.3950 ± 0.2123	0.4479 ± 0.2239	0.5374
PVN	0.8102 ± 0.3112	0.9567 ± 0.3776	0.2300
Inter-FC			
VSN—PVN	0.0294 ± 0.1753	0.0996 ± 0.1211	0.2229
HVN—VSN	0.0499 ± 0.0925	0.1292 ± 0.0841	0.0219*
ssHVN—PVN	0.1108 ± 0.2302	0.3021 ± 0.1776	0.0330*

The marker * indicates significant *p* < 0.05 after FDR correction

## Discussion

In this study, the voxel-based morphometric study demonstrated that cirrhotic patients showed serious gray matter deficits in vision-related regions, including the bilateral lingual gyri, left putamen, right fusiform gyrus, and right calcarine gyrus. Furthermore, a network analysis of brain visual cortices showed the cirrhotic patients suffered from the decreases of internetwork functional connectivity between the HVN and VSN, and that between the HVN and PVN*.* Our findings suggested that functional abnormality of vision-related brain functional networks in patient group compared with HC group.

In the whole-brain VBM analysis, our results suggested the cirrhotic patients suffered from serious reduction in several brain regions, including the bilateral lingual gyri, left putamen, right fusiform gyrus, right calcarine gyrus, and bilateral hippocampus. It was of note that these areas were related with the visual functions. For example, PET studies with colored and moving visual stimulation showed that the lingual gyri and fusiform gyri were critically involved in the color vision [30, 31]. The putamen was reported to be associated with visual attention [32], and a voxel-wise statistical analysis showed that damage in the putamen area was significantly related to deficits in contralateral visual processing speed [33]. The fusiform gyrus has been identified in previous study as important in the visual analysis of human face [34]. Taken together, our VBM result suggested that the vision-related regions showed seriously significant reductions of gray matter volume in patient group without clinical sign of HE.

Based on the structural investigation, we selected brain functional networks within vision-related regions as ROIs from the basis of the atlas template defined by Richiardi et al. [25] for brain functional network analysis. Then, our findings of decreased FC between VSN and HVN, as well as between PVN and HVN, indicate functional impairment within these regions in patient group without clinical sign of HE. The FC reflected the temporal dependency between spatially remote neurophysiological events, which was related to the activity of the underlying neuronal networks [35]. Interestingly, the functional abnormality reflects the impaired functional communication level between remoted cortical regions. Therefore, functional disconnections between HVN and PVN/VSN in patient group showed the impaired co-activation between the neuronal activation patterns of anatomically separated vision-related brain regions compared with HCs.

We speculated that the impaired co-activation might be related to the microstructural alterations in cirrhotic condition. From a neurobiological perspective, some possible mechanisms include, but may not be limited to, both swelling and dysfunction of astrocytes induced by increased concentration of glutamine [36, 37], cytotoxic edema induced by increased intracellular water [38], increased expression of proinflammatory cytokines (including TNF-α and the inter leukins IL-1β and IL-6) [39], and manganese deposition [40]. Thus, the discovery of the current study may supply further neuroimaging evidence for exploring the neural mechanisms of cerebral deficit in the liver cirrhosis without apparent clinical resolution of overt HE, which might provide a basis for further evolution of the disease.

However, several limitations of the present study are noteworthy. First, owing to the limitation of experimental conditions, the psychometric hepatic encephalopathy score (PHES) was not utilized in this study and would be a desirable addition in further studies. In addition, although current results provided the neuroimaging evidence for cerebral lesions in vision-related regions, vision-related examinations should be incorporated in future studies and used to assess the quantitative information of general vision performance. Then, an analysis of the direct correlation would be executed between the clinical characteristics and neuroimaging alterations in cirrhosis without clinical sign of overt HE, which would be of great interest for future investigation.

In conclusion, the current study using voxel-based morphometry showed that the most changed areas were anatomically distributed in the vision-related brain regions. Then, the fMRI-based analysis suggested impaired co-activation in neuronal activity between remote cortical regions in cirrhotic patients without clinical sign of overt HE. Although the physiopathology and clinical relevance of this abnormality could not be ascertained from the current study, our findings provide a basis for further evolution of the disease.
